# Digital Orientation of Health Systems in the Post–COVID-19 “New Normal” in the United States: Cross-sectional Survey

**DOI:** 10.2196/30453

**Published:** 2021-08-16

**Authors:** Jiban Khuntia, Xue Ning, Rulon Stacey

**Affiliations:** 1 CU Business School University of Colorado Denver Denver, CO United States

**Keywords:** post–COVID-19, digital orientation, health systems, digital transformation, digital health, telehealth, telemedicine, COVID-19, impact, insight, cross-sectional, survey, United States, electronic health record, EHR

## Abstract

**Background:**

Almost all health systems have developed some form of customer-facing digital technologies and have worked to align these systems to their existing electronic health records to accommodate the surge in remote and virtual care deliveries during the COVID-19 pandemic. Others have developed analytics-driven decision-making capabilities. However, it is not clear how health systems in the United States are embracing digital technologies and there is a gap in health systems’ abilities to integrate workflows with expanding technologies to spur innovation and futuristic growth. There is a lack of reliable and reported estimates of the current and futuristic digital orientations of health systems. Periodic assessments will provide imperatives to policy formulation and align efforts to yield the transformative power of emerging digital technologies.

**Objective:**

The aim of this study was to explore and examine differences in US health systems with respect to digital orientations in the post–COVID-19 “new normal” in 2021. Differences were assessed in four dimensions: (1) analytics-oriented digital technologies (AODT), (2) customer-oriented digital technologies (CODT), (3) growth and innovation–oriented digital technologies (GODT), and (4) futuristic and experimental digital technologies (FEDT). The former two dimensions are foundational to health systems’ digital orientation, whereas the latter two will prepare for future disruptions.

**Methods:**

We surveyed a robust group of health system chief executive officers (CEOs) across the United States from February to March 2021. Among the 625 CEOs, 135 (22%) responded to our survey. We considered the above four broad digital technology orientations, which were ratified with expert consensus. Secondary data were collected from the Agency for Healthcare Research and Quality Hospital Compendium, leading to a matched usable dataset of 124 health systems for analysis. We examined the relationship of adopting the four digital orientations to specific hospital characteristics and earlier reported factors as barriers or facilitators to technology adoption.

**Results:**

Health systems showed a lower level of CODT (mean 4.70) or GODT (mean 4.54) orientations compared with AODT (mean 5.03), and showed the lowest level of FEDT orientation (mean 4.31). The ordered logistic estimation results provided nuanced insights. Medium-sized (*P*<.001) health systems, major teaching health systems (*P*<.001), and systems with high-burden hospitals (*P*<.001) appear to be doing worse with respect to AODT orientations, raising some concerns. Health systems of medium (*P*<.001) and large (*P*=.02) sizes, major teaching health systems (*P*=.07), those with a high revenue (*P*=.05), and systems with high-burden hospitals (*P*<.001) have less CODT orientation. Health systems in the midwest (*P*=.05) and southern (*P*=.04) states are more likely to adopt GODT, whereas high-revenue (*P*=.004) and investor-ownership (*P*=.01) health systems are deterred from GODT. Health systems of a medium size, and those that are in the midwest (*P*<.001), south (*P*<.001), and west (*P*=.01) are more adept to FEDT, whereas medium (*P*<.001) and high-revenue (*P*<.001) health systems, and those with a high discharge rate (*P*=.04) or high burden (*P*=.003, *P*=.005) have subdued FEDT orientations.

**Conclusions:**

Almost all health systems have some current foundational digital technological orientations to glean intelligence or service delivery to customers, with some notable exceptions. Comparatively, fewer health systems have growth or futuristic digital orientations. The transformative power of digital technologies can only be leveraged by adopting futuristic digital technologies. Thus, the disparities across these orientations suggest that a holistic, consistent, and well-articulated direction across the United States remains elusive. Accordingly, we suggest that a policy strategy and financial incentives are necessary to spur a well-visioned and articulated digital orientation for all health systems across the United States. In the absence of such a policy to collectively leverage digital transformations, differences in care across the country will continue to be a concern.

## Introduction

### Background

The COVID-19 pandemic aggravated the perennial issues of cost, quality, and delivery challenges of health care in the United States. Simultaneously, the COVID-19 pandemic has also opened up newer directions to solve some of these challenges. Digital technologies have come to the forefront in solving many of the challenges. The critical role of technology in fighting the pandemic through effective tracking of the virus across the world is undeniable. Health systems have used existing health record systems along with surveillance and monitoring applications to gather, collate, analyze, and present information to the government to make meaningful and valuable decisions to help in the pandemic. Several technologies played an essential role in informing health systems and frontline health professionals to fight the crisis.

The scope of health information technologies has traditionally been limited to electronic health or medical records, with sporadic examples of data and intelligence-based decision-making using the recorded data. Digital records have been touted to increase the potential to improve health care providers’ efficiency and effectiveness. Key functionalities of electronic health records such as computerized provider order entry for medications, electronic prescribing, or using clinical decision support systems have helped to achieve some of the objectives [1].

The last decade of policies around health information technologies has propelled the adoption of digital records to almost 100%, although the comprehensiveness and meaningful use of these records are still debatable [2]. Nevertheless, the scope of electronic health records to play a transformative role in health care is limited. The fact remains that some health systems are still using a set of basic functionalities rather than fully leveraging more comprehensive functionalities. The result is that these health systems cannot fully shape future decisions and strategies for health care [3].

Industry sectors other than health care have leveraged the transformative potential of digital technologies beyond the capture, archiving, and use of data using digital records only. The increasing prevalence of digital technology is fundamentally transforming how businesses create value. Research has postulated that technology and strategy align together to drive proper digital transformation and ultimately provide a competitive advantage.

In this context, we sought to address the following questions: (1) What are the digital orientations of health systems in the post COVID-2019 new normal? (2) How can such orientations be measured and compared across health systems to provide a systemic evaluation across the United States? (3) What are the factors that may influence the digital orientations of health systems?

With anticipated health care spending to reach US $5.7 trillion by 2026, the time is right to enhance public policy understanding of digital technologies and to build the strategic imperatives around those technologies. However, to change the current standard practices and install improved digital-based technologies into health systems while leveraging these improvements throughout the industry, a comprehensive assessment of the health systems’ digital orientations in 2021 is needed to inform research and practice.

### Literature Review: Digital Orientations

Digital orientation as a strategic direction exhibits superior performance by using and leveraging technology in different ways and through different means while maintaining a view of current and futuristic options [4]. A specific digital orientation can be examined from perspectives such as technology scope and capabilities [4]. Different digital orientations shape the way organizations create and adapt behaviors and resources [5,6], similar to market orientation (eg, [7]) and entrepreneurial orientation (eg, [8]), which have been extensively studied as sources of competitive advantage [9].

Digital orientation with a strategic and futuristic direction will nurture and implement subsequent digitalization initiatives. As a result, digital orientation will create value beyond what is seen as the immediate returns of digital investments [10] and direct to an unprecedented scope and degree of openness, driven by generative and unpredictable processes and contingent on the specific affordances of digital technologies as realized in other sectors [11]. Digital orientation will change the traditional competitive logic to stimulate distinct and novel processes, and managerial and organizational alignment.

The basic step of digital orientation starts with the technologies that support the existing functions of an organization on a day-to-day basis [12,13]. Given that electronic health records have been well disseminated in US health care, the data-driven clinical and administrative decision-making based on mining applications and tools to analyze the data captured along with data available from the records encompass such an orientation. We coin this category of basic orientation as *analytics orientation.*

Another category is *customer-oriented digital technologies* (CODT), which involve technical interfaces through which customers can access services that enable standardized delivery of services [14,15] to provide increased flexibility of access [16,17]. Mobile technologies are an example of tools that provide such access. Social media–integrated tools and applications result in different avenues for the customer to reach these services [18,19].

A set of emerging technologies helps health systems to foster *growth and innovation orientation*. These technologies help to reevaluate and reengineer several business functions, similar to the enterprise resource planning applications [20]. The underlying concept for this orientation is to be innovative in changing business functions and processes, and extending these innovations across partnering businesses to change the value chain. For instance, information exchange with organizations helps to provide just-in-time care effectively while extending care provisions across health systems [21]. Similarly, virtual and remote care models require that the diagnosis and treatment involving physician and patient interactions be redesigned and aligned to newer value-based models relative to the earlier fee-based models.

Finally, *futuristic and experimental digital technologies* (FEDT) are being trialed or experimented with in terms of their potential to change the practice and delivery of health care [22]. These may not be widely disseminated, and the value may not be predictably assessed as is the case for the growth-oriented technologies. Examples of this category would include robotics applications, wearable chips, and tracking devices [23]. Artificial intelligence and machine learning applications are also being introduced to health care, with some value potential, but are waiting for broader dissemination [24].

Delineating the current stage of the four digital orientations described above will aid in guiding strategies and policies in health care. The US health care systems need an overarching digitally enabled strategic orientation to holistically mirror the heightened, transfunctional role of digitalization across the sector. Assessment of these four orientations in 2021 is a first step to guide future actions in this direction.

### Study Aims

Accordingly, the aim of this study was to explore and measure the differences between these four types of orientations in health systems with different characteristics, including size, region, ownership status, teaching status, revenue, number of physicians, hospitals, and other factors. We also explored the impacts of these factors on the levels of the four digital orientations. This study is unique in focusing on the digital orientation of system-wide differences across health systems in the United States. The findings of this study will provide implications for the strategic development of health systems in the post COVID-19 era, and suggest a top-level US health systems digital strategy and plan that can shape the development blueprints for all health systems and the nation.

## Methods

### Data Collection

The effort to assess the digital orientations of health systems is part of a broad project on the climate of health systems undertaken by the Health Administration Research Consortium at the Business School of the University of Colorado Denver. The idea of monitoring health systems emerged from our observations and conversations with several chief executive officers (CEOs) of health systems during the COVID-19 pandemic. The objective was to collect and inform the insights of health systems’ CEOs that will help inform policymakers, practitioners, and academic stakeholders as they collaborate to create ongoing strategies to help the industry respond to this pandemic and prepare for the next crisis.

For the inaugural climate study, a survey questionnaire was developed in December 2020 to collect data from the health systems and scientifically study the climate that health systems face. The survey items were derived from the prior literature with rewording of the questions to fit the health systems context. Inputs were taken from researchers, consultants, and executives with expertise to design the questions. The survey was validated using a scientific process of experts’ evaluations and was pilot-tested with five top executives who are part of the Health Administration Program Advisory Board. The survey questionnaire was revised and finalized in January 2021 [25].

We compiled a contacts list of CEOs of a total of 624 health systems across the United States using information from multiple sources, contacts, professional collections, websites, and annual reports. We mounted the survey instrument on a professional survey platform. We mapped the emails to the platform to create unique trackable links for each health system. We sent the invitation and solicitation emails to the CEOs in multiple rounds between January 25 and March 2, 2021. Along with this, the authors called several CEOs and solicited completion of the survey instrument either online or in paper format. The researchers also requested CEOs who had participated in the survey to share the link with other CEO colleagues. We received a total of 148 responses, with a 24% response rate. We could not use 13 incomplete responses, leaving 135 final usable responses for analysis.

The size of the 135 health systems represented in this survey varied from 1 to 18 hospitals with 176 to 75,000 employees. The annual revenue in 2020 of the health systems ranged from US $0.7 million to US $14 billion. The health systems aggregately represent US $0.3 trillion in revenues with 1.1 million employees across the United States.

We then matched the survey dataset with the secondary data collected from the Agency for Healthcare Research and Quality Hospital Compendium to obtain a better and more complete picture of the health systems. Finally, we obtained a dataset of 124 health systems that are located across the United States. We analyzed this combined dataset to report several insights in this study.

### Variables and Measures

Table 1 shows the description of the variables used in this study. The primary dependent variables were the four digital orientations: (1) analytics-oriented digital technologies (AODT), (2) CODT, (3) growth and innovation–oriented digital technologies (GODT), and (4) FEDT.

**Table 1:** Description of variables, including survey questions and coding scheme.

Variable						Description
**Survey question: “To what extent do you consider the following digital technologies to be important and creating value for your health systems?”^a^**						
	AODT				Analytics-oriented digital technologies, including data mining and analysis, data-driven administrative decision-making, and data-driven clinical decision-making (Cronbach α=.77)	
	CODT				Customer-oriented digital technologies, including mobile technologies for customer engagement (eg, social media tools, applications, and integration), rendering higher customer-oriented services (Cronbach α=.57)	
	GODT				Growth and innovation–oriented digital technologies, including reengineering several business functions, providing innovation potential within the organization, and providing innovation capacity in collaboration with external organizations (Cronbach α*=*.57)	
	FEDT				Futuristic and experimental digital technologies, including virtual monitoring using wearables, chips, and tracking devices; robotics applications in health care; and artificial intelligence and machine learning (Cronbach α*=*.91)	
**Contingent variables**						
	**SIZE**				Total beds managed by the health system across all hospitals, reported by the Agency for Healthcare Research and Quality (AHRQ) Hospital Compendium	
		SIZE_B-SMALL		<100 beds		
		SIZE_B-MEDIUM		100-400 beds		
		SIZE_B-LARGE		>400 beds		
	**REGION**				Primary location of the health system in the United States, following the Census Bureau categorization	
		REGION-NE		Northeast		
		REGION-MW		Midwest		
		REGION-SOUTH		South		
		REGION-WEST		West		
	**TEACHING**				Teaching status of a health system	
		TEACHING-NON		nonteaching		
		TEACHING-MINOR		minor teaching		
		TEACHING-MAJOR		major teaching		
	**REVENUE**				Annual revenue (US $) of the health system across all hospitals	
		REVENUE-LOW		<2 billion		
		REVENUE-MEDIUM		2-5 billion		
		REVENUE-HIGH		>5 billion		
	HIGH-DSH-HOSP				The health system includes at least one Disproportionate Share Hospital (DSH) (1=yes, 0=no), which refers to a hospital that serves a significantly disproportionate number of low-income patients and receives payments from the Centers for Medicaid and Medicare Services of the United States to cover the costs of providing care to uninsured patients.	
	**BURDEN**				This reflected the uncompensated care, as an overall measure of hospital care provided for which no payment was received from the patient or insurer.	
		HIGH-BURDEN-SYS		Health system–wide uncompensated care burden flag (1=yes, 0=no)		
		HIGH-BURDEN-HOSP		The health system includes at least one high uncompensated care burden hospital (1=yes, 0=no)		
	OWNERSHIP				Predominantly investor-owned hospitals (1=yes, 0=no)	
	PHYSICIANS				Number of physicians in the health system as reported by the AHRQ Hospital Compendium.	
	HOSPITALS				Number of hospitals in the health system as reported by the AHRQ Hospital Compendium	

^a^All questions were measured using a 7-point Likert scale (1=strongly disagree to 7=strongly agree).

These four variables were measured based on a 7-point Likert scale of relevant items. The reliability was tested using Cronbach α, which was higher than the acceptable threshold of .50 for reflective items and measures used in this study [26].

The independent variables of this study covered several categories: size, region, teaching status, revenue, and other system characteristics. These variables were coded to reflect the characteristics of a health system, which may influence its digital orientations (see Table 1). In summary, three size variables were used to measure the number of beds across a health system (SIZE_B-SMALL, SIZE_B-MEDIUM, SIZE_B-LARGE); four region variables were used to reflect the location of a health system (REGION-NE, REGION-MW, REGION-SOUTH, REGION-WEST), there were three teaching status–related variables (TEACHING-NON, TEACHING-MINOR, TEACHING-MAJOR), and three revenue variables were used to measure the annual revenue of a health system (REVENUE-LOW, REVENUE-MEDIUM, REVENUE-HIGH). Other variables included those related to the existence of Disproportionate Share Hospital (DSH) patients (HIGH-DSH-HOSP), uncompensated care burden (HIGH-BURDEN-SYS and HIGH-BURDEN-HOSP), ownership status (OWNERSHIP), number of physicians (PHYSICIANS), and number of hospitals (HOSPITALS).

### Sample Statistics

The descriptive statistics and pairwise correlations among the key variables used in this study are presented in Table 2 and Table 3. As shown in Table 2, health systems have a lower level of customer or growth orientations compared to AODT orientations, and showed the least amount of FEDT.

In addition, to make sure there is no nonresponse bias, we compared the characteristics of responding and nonresponding health systems. The detailed comparisons are provided in Table 4. The *t* test results for all comparisons indicated no significant difference between respondents and nonrespondents.

**Table 2:** Summary statistics (N=124).

Variable^a^	Mean (SD)	Minimum	Maximum
AODT^b^	5.03 (1.37)	2	6.67
CODT^c^	4.70 (1.35)	2.33	7
GODT^d^	4.54 (1.23)	2.33	7
FEDT^e^	4.31 (1.54)	1	7
SIZE_B-SMALL	0.09 (0.28)	0	1
SIZE_B-MEDIUM	0.37 (0.49)	0	1
SIZE_B-LARGE	0.54 (0.50)	0	1
REGION-NE	0.22 (0.42)	0	1
REGION-MW	0.24 (0.43)	0	1
REGION-SOUTH	0.35 (0.48)	0	1
REGION-WEST	0.18 (0.38)	0	1
TEACHING-NON	0.30 (0.46)	0	1
TEACHING-MINOR	0.48 (0.50)	0	1
TEACHING-MAJOR	0.22 (0.41)	0	1
REVENUE-LOW	0.61 (0.49)	0	1
REVENUE-MEDIUM	0.23 (0.43)	0	1
REVENUE-HIGH	0.15 (0.35)	0	1
HIGH-DSH-HOSP	0.33 (0.47)	0	1
HIGH-BURDEN-SYS	0.20 (0.40)	0	1
HIGH-BURDEN-HOSP	0.30 (0.46)	0	1
OWNERSHIP	0.02 (0.13)	0	1
PHYSICIANS	1.84 (0.80)	1	3
HOSPITALS	1.50 (0.77)	1	3

^a^See Table 1 for variable and code definitions.

^b^AODT: analytics-oriented digital technologies

^c^CODT: customer-oriented digital technologies.

^d^GODT: growth and innovation–oriented digital technologies.

^e^FEDT: futuristic and experimental digital technologies.

**Table 3:** Pairwise correlations (Pearson *r* and *P* values) among key variables (N=124).

Variable^a^				1		2		3		4		5		6		7		8		9		10		11		12		13		14		15		16		17		18		19
**1. AODT^b^**																																								
		*r*	1.00		0.70		0.06		–0.15		–0.13		0.03		–0.06		–0.02		0.16		–0.01		–0.11		0.04		–0.07		0.07		–0.03		–0.05		0.01		–0.05		0.06	
		*P*	—^c^		<.001		.52		.09		.14		.75		.50		.81		.08		.95		.23		.65		.41		.42		.75		.56		.88		.62		.51	
**2. CODT^d^**																																								
		*r*	0.70		1.00		–0.13		0.01		–0.07		–0.05		–0.01		–0.16		0.24		–0.01		–0.15		–0.13		–0.02		–0.02		0.08		–0.14		0.01		–0.10		–0.04	
		*P*	<.001		—		.16		.94		.46		.58		.90		.07		.01		.91		.09		.15		.85		.82		.37		.11		.89		.16		.63	
**3. GODT^e^**																																								
		*r*	0.06		–0.13		1.00		0.43		–0.08		0.13		0.13		0.03		0.001		–0.01		0.10		0.15		–0.02		–0.03		0.06		0.11		–0.21		0.08		0.08	
		*P*	.52		.16		—		<.001		.40		.15		.15		.71		.99		.92		.29		.09		.82		.77		.50		.23		.02		.37		.36	
**4. FEDT^f^**																																								
		*r*	–0.15		0.01		0.43		1.00		0.01		0.06		0.10		–0.01		0.16		–0.08		0.07		0.003		0.0001		–0.15		0.22		–0.01		–0.15		0.04		–0.07	
		*P*	.09		.94		<.001		—		.92		.54		.28		.95		.07		.38		.44		.98		.99		.10		.01		.94		.09		.63		.41	
**5. SIZE_B-MED**																																								
		*r*	–0.13		–0.07		–0.08		0.01		1.00		–0.83		–0.08		–0.01		–0.05		–0.10		–0.16		–0.27		–0.32		–0.08		–0.01		–0.21		0.03		–0.60		–0.46	
		*P*	.14		.46		.40		.92		—		<.001		.36		.90		.58		.29		.07		.003		<.001		.39		.89		.02		.71		<.001		<.001	
**6. SIZE_B-LARGE**																																								
		*r*	0.03		–0.05		0.13		0.06		–0.83		1.00		–0.01		0.11		0.005		0.26		0.25		0.36		0.38		0.17		0.06		0.28		–0.14		0.75		0.54	
		*P*	.75		.58		.15		.54		<.001		—		.93		.23		.96		.003		.005		<.001		<.001		.06		.51		.001		.12		<.001		<.001	
**7. REGION-MW**																																								
		*r*	–0.06		–0.01		0.13		0.10		–0.08		–0.01		1.00		–0.42		–0.26		–0.01		–0.12		–0.09		0.09		–0.20		–0.10		0.002		–0.07		0.02		0.02	
		*P*	.50		.90		.15		.28		.36		.93		—		<.001		.003		.91		.20		.32		.33		.03		.29		.98		.42		.83		.79	
**8. REGION-SOUTH**																																								
		*r*	–0.02		–0.16		0.03		–0.01		–0.01		0.11		–0.42		1.00		–0.34		0.07		0.02		0.15		–0.02		0.12		0.17		0.22		0.17		0.002		0.04	
		*P*	.81		.07		.71		.95		.90		.23		<.001		—		<.001		.44		.85		.10		.84		.17		.05		.02		.06		.98		.63	
**9. REGION-WEST**																																								
		*r*	0.16		0.24		0.001		0.16		–0.05		0.005		–0.26		–0.34		1.00		–0.02		–0.04		–0.06		–0.01		0.12		0.08		0.02		–0.06		–0.04		–0.03	
		*P*	.08		.01		.99		.07		.58		.96		.003		<.001		—		.83		.66		.53		.89		.18		.36		.82		.51		.67		.76	
**10. TEACHNG-MINOR**																																								
		*r*	–0.01		–0.01		–0.01		–0.08		–0.10		0.26		–0.01		0.07		–0.02		1.00		–0.50		0.12		0.07		0.05		–0.12		0.08		0.01		0.19		0.26	
		*P*	.95		.91		.92		.38		.29		.003		.91		.44		.83		—		<.001		.18		.47		.57		.20		.35		.95		.03		.003	
**11. TEACHNG-MAJOR**																																								
		*r*	–0.11		–0.15		0.10		0.07		–0.16		0.25		–0.12		0.02		–0.04		–0.50		1.00		0.12		0.17		0.34		0.03		0.13		–0.07		0.38		0.06	
		*P*	.23		.09		.29		.44		.07		.005		.20		.85		.66		<.001		—		.17		.06		<.001		.77		.16		.46		<.001		.48	
**12. REVENUE-MED**																																								
		*r*	0.04		–0.13		0.15		0.003		–0.27		0.36		–0.09		0.15		–0.06		0.12		0.12		1.00		–0.23		–0.06		–0.04		0.14		–0.07		0.26		0.29	
		*P*	.65		.15		.09		.98		.003		<.001		.32		.10		.53		.18		.17		—		.01		.48		.66		.12		.44		.004		.001	
**13. REVENUE-HIGH**																																								
		*r*	–0.07		–0.02		–0.02		0.0001		–0.32		0.38		0.09		–0.02		–0.01		0.07		0.17		–0.23		1.00		0.15		–0.04		–0.02		–0.05		0.51		0.30	
		*P*	.41		.85		.82		.99		<.001		<.001		.33		.84		.89		.47		.06		.01		—		.10		.69		.84		.56		<.001		<.001	
**14. HIGH-DSH-HOSP**																																								
		*r*	0.07		–0.02		–0.03		–0.15		–0.08		0.17		–0.20		0.12		0.12		0.05		0.34		–0.06		0.15		1.00		–0.01		0.18		0.05		0.23		0.17	
		*P*	.42		.82		.77		.10		.39		.06		.03		.17		.18		.57		<.001		.48		.10		—		.90		.05		.61		.01		.06	
**15. HIGH-BURD-SYS**																																								
		*r*	–0.03		0.08		0.06		0.22		–0.01		0.06		–0.10		0.17		0.08		–0.12		0.03		–0.04		–0.04		–0.01		1.00		0.42		–0.06		–0.10		–0.20	
		*P*	.75		.37		.50		.01		.89		.51		.29		.05		.36		.20		.77		.66		.69		.90		—		<.001		.48		.27		.03	
**16. HIGH-BURD-HOSP**																																								
		*r*	–0.05		–0.14		0.11		–0.01		–0.21		0.28		0.002		0.22		0.02		0.08		0.13		0.14		–0.02		0.18		0.42		1.00		–0.08		0.18		0.31	
		*P*	.56		.11		.23		.94		.02		.001		.98		.02		.82		.35		.16		.12		.84		.05		<.001		—		.36		.05		<.001	
**17. OWNERSHIP**																																								
		*r*	0.01		0.01		–0.21		–0.15		0.03		–0.14		–0.07		0.17		–0.06		0.01		–0.07		–0.07		–0.05		0.05		–0.06		–0.08		1.00		0.18		0.31	
		*P*	.88		.89		.02		.09		.71		.12		.42		.06		.51		.95		.46		.44		.56		.61		.48		.36		—		.55		.36	
**18. PHYSICIANS**																																								
		*r*	–0.05		–0.10		0.08		0.04		–0.60		0.75		0.02		0.002		–0.04		0.19		0.38		0.26		0.51		0.23		–0.10		0.18		–0.05		1.00		–0.08	
		*P*	.62		.16		.37		.63		<.001		<.001		.83		.98		.67		.03		<.001		.004		<.001		.01		.27		.05		.55		—		<.001	
**19. HOSPITALS**																																								
		*r*	0.06		–0.04		0.08		–0.07		–0.46		0.54		0.02		0.04		–0.03		0.26		0.06		0.29		0.30		0.17		–0.20		0.31		–0.08		0.57		1.00	
		*P*	.51		.63		.36		.41		<.001		<.001		.79		.63		.76		.003		.48		.001		<.001		.06		.03		<.001		.36		<.001		—	

^a^See Table 1 for variable and code definitions.

^b^Not applicable.

^c^AODT: analytics-oriented digital technologies

^d^CODT: customer-oriented digital technologies.

^e^GODT: growth and innovation–oriented digital technologies.

^f^FEDT: futuristic and experimental digital technologies.

**Table 4:** Characteristics of responding and nonresponding health systems.

Characteristics		Respondents, n (%) (N=124)	Nonrespondents, n (%) (N=511)	*t* value (*df*)
**Size**				
	Small (6-99 beds)	11 (8.9)	43 (8.4)	–0.19 (50)
	Medium (100-399 beds)	45 (36.3)	212 (41.5)	–0.56 (254)
	Large (≥400 beds)	68 (54.9)	256 (50.1)	1.41 (325)
**Region**				
	Northeast	27 (21.8)	118 (23.1)	0.07 (141)
	Midwest	30 (24.2)	133 (26.0)	0.55 (162)
	South	45 (36.3)	167 (32.7)	–0.48 (214)
	West	22 (17.7)	93 (18.2)	–0.12 (112)
**Physicians^a^**				
	Small (51-199 physicians)	50 (40.3)	189 (37.0)	–0.74 (238)
	Medium (200-999 physicians)	41 (33.1)	204 (39.9)	–0.69 (243)
	Large (≥1000 physicians)	33 (26.6)	118 (23.1)	1.53 (150)
**Hospitals^a^**				
	Small (1-3 hospitals)	83 (66.9)	338 (66.1)	–1.27 (420)
	Medium (4-6 hospitals)	20 (16.1)	66 (12.9)	–0.02 (85)
	Large (≥7 hospitals)	21 (16.9)	107 (20.9)	0.81 (126)
**Ownership status**				
	Investor-owned	3 (2.4)	15 (2.9)	–0.85 (15)
	Noninvestor-owned	121 (97.6)	496 (97.1)	0.85 (616)
**Teaching status**				
	Major teaching	29 (23.3)	138 (27.0)	–0.15 (186)
	Minor teaching	58 (46.8)	225 (44.0)	–0.61 (280)
	Nonteaching	37 (29.8)	148 (29.0)	0.85 (163)

^a^The numbers of physicians and hospitals are presented in this table in different categories for easy comparison across respondents and nonrespondents.

### Statistical Analysis

We used ordered logit regressions to estimate the relationships of the four digital orientations to specific hospital characteristics. All four dependent variables are ordinal variables to drive the decision for ordered logit regressions. This approach does not assume equal intervals between levels in the dependent variable. The ordered logit model is as follows:

Y_i_*=*βX_i_*+*e_i_*

Where Y_i_* is the propensity of respondents to indicate higher levels of the four digital orientations (ie, AODT, CODT, GODT, FEDT), *X_i_* is a set of explanatory variables, β a vector of parameters, and *e_i_* represents disturbances.

Rather than observing Y_i_*, we observed the ordinal dependent variable *Y_i_* depending on the values of thresholds or cut-off points *τ_m_*_–1_ and *τ_m_.* The probability distribution of *Y_i_* is given as follows:

Pr(*Y_i_*=*m*|**X***_i_*=*F*(*τ_m_*–X*β*) – *F*(*τ_m_*_–1_–X*β*)

where


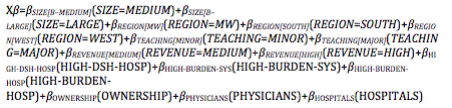


## Results

Table 5 shows the results of the ordered logit model estimation. Each column presents the results for each of the four digital orientations.

First, we found that the two size variables have a significantly negative association with AODT. In particular, the medium size variable showed high statistical significance at *P*<.001. This suggests that smaller-sized health systems tend to adopt analytics and intelligence-oriented digital technologies. Based on the marginal effects analysis, we found that compared to small-sized health systems, there is a 0.145 decrease in the probability of adopting AODT by medium-sized health systems.

We found a significant and negative relationship between major teaching health systems and AODT (*P*<.001), indicating that compared to major teaching health systems, nonteaching health systems have a greater orientation toward AODT. The marginal effects analysis suggested a 0.123 decrease in the probability of adopting AODT in major teaching health systems than in the nonteaching health systems.

A high-burden hospital also had a significant and negative impact on AODT (*P*<.001). This result indicates that a health system without a high uncompensated care burden hospital is more likely to use analytics technologies. We also examined the marginal effects of this variable. The result indicated a 0.039 decrease in the probability of using AODT by a health system with at least one high uncompensated care burden hospital.

**Table 5:** Ordered logit model estimation results.^a^

Variables^b^	AODT^c^ (pseudo-R^2^=0.027)		CODT^d^ (pseudo-R^2^=0.035)		GODT^e^ (pseudo-R^2^=0.031)			FEDT^f^ (pseudo-R^2^=0.048)	
	Coefficient (SE)	*P* value	Coefficient (SE)	*P* value	Coefficient (SE)	*P* value	Coefficient (SE)		*P* value
SIZE_B-MEDIUM	–1.283 (.181)	<.001	–1.510 (.290)	<.001	.005 (.305)	.99	.863 (.495)		.08
SIZE_B-LARGE	–.804 (.407)	.05	–1.058 (.454)	.02	.123 (.390)	.75	.707 (.558)		.21
REGION-MW	.139 (.528)	.79	–.078 (.689)	.91	1.005 (.507)	.05	1.365 (.204)		<.001
REGION-SOUTH	–.009 (.355)	.98	–.386 (.504)	.44	.745 (.370)	.04	1.235 (.174)		<.001
REGION-WEST	.391 (.446)	.38	.604 (.560)	.28	.630 (.474)	.18	2.074 (.817)		.01
TEACHING-MINOR	–.182 (.820)	.82	.145 (.649)	.82	–.072 (.874)	.93	.211 (.920)		.82
TEACHING-MAJOR	–1.004 (.218)	<.001	–.410 (.225)	.07	.268 (1.038)	.80	.879 (.801)		.27
REVENUE-MEDIUM	.341 (.526)	.52	–.487 (.365)	.18	.430 (.877)	.62	–.255 (.061)		<.001
REVENUE-HIGH	–.025 (.258)	.92	–.166 (.084)	.05	–.357 (.124)	.004	–.245 (.062)		<.001
HIGH-DSH-HOSP	.612 (.403)	.13	.220 (.224)	.33	–.045 (.503)	.93	–.891 (.435)		.04
HIGH-BURDEN-SYS	.562 (.697)	.42	.980 (.644)	.13	.200 (.208)	.34	1.018 (.347)		.003
HIGH-BURDEN-HOSP	–.376 (.087)	<.001	–.880 (.250)	<.001	–.016 (.202)	.94	–.784 (.281)		.005
OWNERSHIP	–.280 (1.299)	.83	.153 (2.113)	.94	–4.934 (1.974)	.01	–1.523 (1.356)		.26
PHYSICIANS	–.080 (.054)	.14	–.142 (.104)	.17	.048 (.662)	.94	.342 (.260)		.19
HOSPITALS	.180 (.205)	.38	.288 (.174)	.10	.068 (.118)	.57	–.131 (.136)		.34

^a^The results of the cut-off points are omitted for brevity.

^b^See Table 1 for descriptions of the variable codes.

^c^AODT: analytics-oriented digital technologies.

^d^CODT: customer-oriented digital technologies.

^e^GODT: growth and innovation–oriented digital technologies.

^f^FEDT: futuristic and experimental digital technologies.

We found a significant and negative relationship between the CODT orientation and medium size (*P*<.001) as well as large size (*P*=.05), indicating that smaller-sized health systems are apt to adopt CODT. The marginal effects analysis showed that the probability changes for these two factors were 0.130 and 0.073, respectively.

The significant and negative relationships between major teaching (*P*=.07), high revenue (*P*=.05), and inclusion of a high-burden hospital (*P*<.001) and CODT suggest that nonteaching health systems, low-revenue health systems, and health systems that do not have high-burden hospitals are more likely to adopt digital technologies for their customers. The marginal effects for these three variables were 0.032, 0.013, and 0.072, respectively.

Compared with health systems in the northeast, health systems in the midwest (*P*=.05) and south (*P*=.04) were found to be more likely to adopt GODT. These results reveal the influence of health systems’ location on their orientation to GODT. More specifically, marginal effects analysis indicated a 0.006 and 0.005 increase in the probability of adopting GODT for health systems in the midwest and southern states, respectively.

Table 5 also shows negative relationships between GODT and high revenue (*P*=.004) as well as ownership (*P*=.01), suggesting that low-revenue health systems and health systems that are owned by noninvestors tend to use GODT. According to the marginal effects, the more specific tendency changes were 0.003 and 0.477 for these two variables, respectively.

Table 5 also shows that the regions of health systems have significant impacts on the FEDT orientation, with health systems in the midwest (*P*<.001), south (*P*<.001), and west (*P*=.01) being more likely to adopt FEDT than those in the northeast. The changes in the marginal effects were 0.006, 0.006, and 0.007, respectively.

There were positive relationships between FEDT and medium size (*P*=.08) and system-wide burden (*P*=.003), suggesting that medium-sized health systems and health systems that have a high system-wide uncompensated care burden tend to adopt FEDT. The changes in the probability of adopting FEDT were the same for these two variables (0.004).

By contrast, there were negative relationships between FEDT and medium revenue (*P*<.001), high revenue (*P*<.001), the inclusion of hospitals with DSH patients (*P*=.04), and inclusion of high-burden hospitals (*P*=.005). These results indicate that low-revenue health systems, and health systems without a high DSH patient percentage hospital and no high uncompensated care burden hospitals are more inclined to use FEDT. According to the marginal effects analysis, these health systems indicate an increase of 0.001, 0.001, 0.006, and 0.005, respectively, in the probability of adopting FEDT.

## Discussion

### Principal Findings

This study first explored the digital orientations of health systems across the United States and then examined the factors that may influence the digital orientations of health systems, comparing across the current analytics and customer-oriented technologies, and the growth and futuristic-oriented technologies [27]. The main findings suggest that (1) health systems in the midwest and southern states, along with low-revenue and noninvestor-owned health systems have growth or futuristic digital orientations (ie, GODT or FEDT); and (2) small-sized, nonteaching, and less burdened health systems are still focusing on current digital technologies such as analytics or customer-oriented technologies (ie, AODT or CODT).

The first set of results suggests the impacts of size, teaching status, and burden of a health system on its digital orientations. More specifically, the smaller-sized health systems are more likely to adopt analytics and customer-oriented digital technologies. Plausibly, smaller health systems are constrained by the complexities of digital technologies to explore advanced digital technologies such as artificial intelligence and robotics [28]. Smaller health systems may not have a research and development team or an independent information technology department to steer and improve future technologies and align them to create value.

Second, the findings show that nonteaching health systems tend to focus more on analytics and customer-oriented digital technologies. It is widely known that teaching hospitals are somehow focused on more cutting-edge technologies and experiment on the future technologies compared with nonteaching hospitals that have limited access to such opportunities. Accordingly, the variation in the strategic vision to guide digital orientations is apparent [29,30].

Third, health systems including hospitals with a lower uncompensated care burden are apt to choose AODT or CODT. According to a prior study, uncompensated care decreased at hospitals when there was Medicaid expansion [31]. A low uncompensated care burden health system presumably has higher revenue, and has no strong motivation to be future-thinking. In other words, such health systems are satisfied with the revenue from traditional care avenues through the current basic analytics and customer-oriented digital technologies.

The second set of findings highlights the role of location, revenue, and ownership status of a health system based on its adoption of digital technologies. First, we found that health systems in the midwest and south are more growth and futuristic-oriented. An explanation for this may be that while health systems in the northeast had advanced with respect to records-based digital technologies, systems located in the midwest and south have lagged in this transition [32,33]. While realizing the value potential, systems in the midwest and south may be trying to make up for the lost time to gain a competitive advantage.

Similarly, health systems with a low revenue are also more likely to adopt futuristic and growth-oriented digital technologies. Although it appears counterintuitive, leaders of low-revenue systems have strong motivation to explore and leverage futuristic digital technology to grow rather than risk failure by sustained low revenue. In other words, low-revenue systems are aspiring that the futuristic technologies will help them to be efficient and cost-effective on the digital transformative path.

Third, the results show that noninvestor-owned health systems have higher probabilities of adopting growth and futuristic digital technologies. The reason may be related to one of the following two aspects. On the one hand, it may be that investor-owned hospitals have already spent resources on state-of-the-art digital technologies, and further investment is duplicative. On the other hand, investor-owned systems may be risk-averse given that quarterly earnings are rarely driven by high-cost digital investment [34,35]. Nevertheless, noninvestor-owned health systems find themselves in a unique position to take advantage of investor-owned health systems’ slow adoption of future-oriented digital technologies.

### Implications

These findings have several practice and policy implications. There are strong indications that small-sized and low-revenue health systems need financial incentives to bridge the digital gap. Although their aspirations are high, current revenues may not allow the investment needed to create a competitive advantage. On a similar note, it is possible that health systems’ aspirations will end in unmet expectations unless those expectations are used to guide the health system through effective adoption and utilization of appropriate digital technologies.

Earlier failures of electronic health record implementations by several health systems indicate that digital technology adoption and implementation is a risky venture. Given past failures, evidence suggests that some health systems are either not disciplined enough or financially prepared for such an implementation. We suggest that policymakers pay attention to these past failures and formulate a well-orchestrated, incentive-based approach for these health systems to succeed in the future.

A point of concern here is the observed significant variations of health systems’ digital orientations. Although it may be realistic to expect that some variations are unavoidable, such significant variations are of some concern, particularly when all health systems are engaged in similar operations, businesses, and service delivery approaches. This again points to the lack of consensus and specific public policy in the health care sector regarding the development of digital technologies across the health system. It is clear that a top-level US health systems digital strategy and plan, driving all health systems with similar implementation criteria is desperately needed.

Once an appropriate public policy is in place, we believe that the market will drive relevant training opportunities, improve organizational capabilities, and focus the attention of CEOs necessary to drive regional developments. Additionally, we believe that such a process will successfully drive financial, operational, and strategic support for nondigital health systems that cannot thrive in the absence of such support.

At present, it appears that there are only a few senior leaders of health systems who can, without hesitation, state that their system has a health care technology plan and program. Some wonder if they even have a program at all. As these leaders can adapt to a more significant industry-wide policy of digital technology, their systems will be better positioned to move toward the future to help overcome employee-level resistance to these changes.

### Limitations and Directions for Future Research

We acknowledge some limitations of this study. First, although we examined the impacts of revenue on digital orientations, we could not capture the actual digital expenditures, which is a more significant factor for health systems’ digital options. In the future, we plan to collect data to reflect this factor.

Furthermore, there are several significant barriers to adopting futuristic digital technologies, such as security concerns. Future studies may focus on how these barriers and orientations are aligned.

We also recognize that the underlying tone in this study is that the growth and futuristic orientation is more important than the customer and analytic orientation for health systems, following prior research [4,27]. However, in the current US health care industry, we acknowledge that customer orientation and analytics-driven intelligence also play significant roles in improving quality and efficiency while reducing care delivery costs. Independent of this perspective, a future study may correlate the digital technology orientations with the performance of health systems to justify this assumption. Moreover, we have only focused on the influences of objective factors on digital orientations in this study due to the nature of using secondary data. Future studies may consider other subjective factors such as senior leadership support and strategic alignment–relevant factors.

### Conclusions

The challenges and uncertainties that the COVID-19 pandemic presented to health systems in the United States were unprecedented. The pandemic propelled the transformative and disruptive powers of digitalization to the forefront. The unprecedented surge of telehealth with remote and virtual care reshaped delivery models, which changed the relationship between patients and care providers. Further, the pandemic relatively quickly reshaped the acceptance of virtual technology. More than ever before, health care was provided virtually, and patients who used to have to come to a hospital or clinic were free from that burden. Given this change, senior leaders need to understand the digital orientations in their health systems to address the challenges and prepare for the uncertainties.

Almost all health systems have adopted customer-facing digital technologies to enable remote and virtual care deliveries. Indeed, several health systems have analytics-driven decision-making capabilities. Nevertheless, not many health systems use technologies for workflow alignments to spur innovation and futuristic growth. On the one hand, smaller-sized, nonteaching, and low-burdened health systems tend to adopt analytics and customer-oriented digital technologies. The rationale for their choice may be financial constraints, lack of capability, and lack of support with respect to policy or technical support.

Finally, health systems in the midwest and south, along with low-revenue and noninvestor-owned health systems are more likely to adopt futuristic and growth-oriented digital technologies. The underlying reasons can be very complex, but this finding indicates the development pattern regarding location, financial performance, and ownership status. Some traditionally underrepresented health systems are making efforts to grow by leveraging disruptive digital technologies. While this is excellent progress, such efforts need to be supported at the highest echelons of the policy level. With guidance, these policies can better ensure that future failures are avoided.

The response to the disruption of the COVID-19 pandemic highlights the significance of digital technologies. In the post COVID-19 era, we believe that more and more health systems will see the value of digital transformation. However, some health systems may fall back in this process due to resource constraints, including tangible resources such as budget and intangible resources such as information technology capabilities [36]. It is crucial to provide policy and technical assistance to support the future-oriented digital transformation efforts in health systems. We give the clarion call to form a top-level US health systems digital strategy and plan to shape the development blueprints for all health systems and the nation.

## Abbreviations

AODT: analytics-oriented digital technologies
CEO: chief executive officer
CODT: customer-oriented digital technologies
DSH: Disproportionate Share Hospital
FEDT: futuristic and experimental digital technologies
GODT: growth and innovation–oriented digital technologies
